# Seasonal Influenza A Virus in Feces of Hospitalized Adults

**DOI:** 10.3201/eid1711.110205

**Published:** 2011-11

**Authors:** Martin C.W. Chan, Nelson Lee, Paul K.S. Chan, K.F. To, Rity Y.K. Wong, Wing-Shan Ho, Karry L.K. Ngai, Joseph J.Y. Sung

**Affiliations:** The Chinese University of Hong Kong, Hong Kong Special Administrative Region, People’s Republic of China

**Keywords:** influenza, viruses, seasonal influenza A, gastrointestinal, fecal virus detection, Hong Kong, adults

## Abstract

In a cohort of hospitalized adults with seasonal influenza A in Hong Kong, viral RNA was frequently (47%) detected in stool specimens. Viable virus was rarely isolated. Viral RNA positivity had little correlation with gastrointestinal symptoms and outcomes. In vitro studies suggested low potential for seasonal influenza viruses to cause direct intestinal infections.

Although influenza predominantly causes respiratory diseases, gastrointestinal signs such as diarrhea and vomiting are not uncommonly reported, particularly among young, hospitalized children (8%–38%) and immunocompromised persons (*1**–**3*). Influenza virus RNA has been detected in feces, but its role is unknown (*4**–**7*). We investigated fecal viral RNA shedding in a large cohort of hospitalized adults with seasonal influenza A in Hong Kong Special Administrative Region, People’s Republic of China. The potential of seasonal influenza viruses to cause direct intestinal infections was examined.

## The Study

We conducted a prospective, observational study among adults hospitalized with laboratory-confirmed seasonal influenza A infections during 2006–2009. Hospital admission, diagnosis, and management procedures have been described (*8*). Briefly, patients were admitted if severe symptoms, respiratory or cardiovascular complications, or exacerbations of underlying conditions developed. When the patients sought care, nasopharyngeal aspirates (NPAs) were collected for diagnosis by using immunofluorescence assay or reverse transcription PCR. Patients with confirmed influenza A were recruited if they were >18 years of age and sought care within 1 week of illness onset. Patients with pandemic (H1N1) 2009 virus infections were excluded and reported separately (*9*).

After providing written, informed consent, patients were asked to submit 1 stool specimen for viral RNA detection during hospitalization, regardless of gastrointestinal symptoms. Clinical information was prospectively recorded (*8*). For comparison, fecal shedding of respiratory syncytial virus (RSV) and parainfluenza virus (PIV) were studied during a 10-month period by using a similar approach. Ethical approval for the study was obtained from the institutional review boards of The Chinese University of Hong Kong.

All stool specimens were subjected to influenza viral RNA detection by using quantitative real-time reverse transcription PCR targeting the matrix gene (*6*). If positive, virus subtyping was performed by using H1- and H3-specific conventional PCRs. Freshly collected stool specimens during 1 seasonal peak were simultaneously subjected to viral RNA detection and virus isolation by using MDCK cells. Detailed methods for fecal detection of influenza viruses and RSV and PIV are provided in the Technical Appendix.

Lectin histochemical analysis and double immunofluorescence staining were used to study the distribution of influenza virus receptors on human small and large intestinal tissues. An in vitro virus binding study on intestinal tissues was also performed by using inactivated human virus isolates of subtypes H1N1 (A/HongKong/CUHK-13003/2002) and H3N2 (A/HongKong/CUHK-22910/2004) (Technical Appendix).

**Figure 1:**
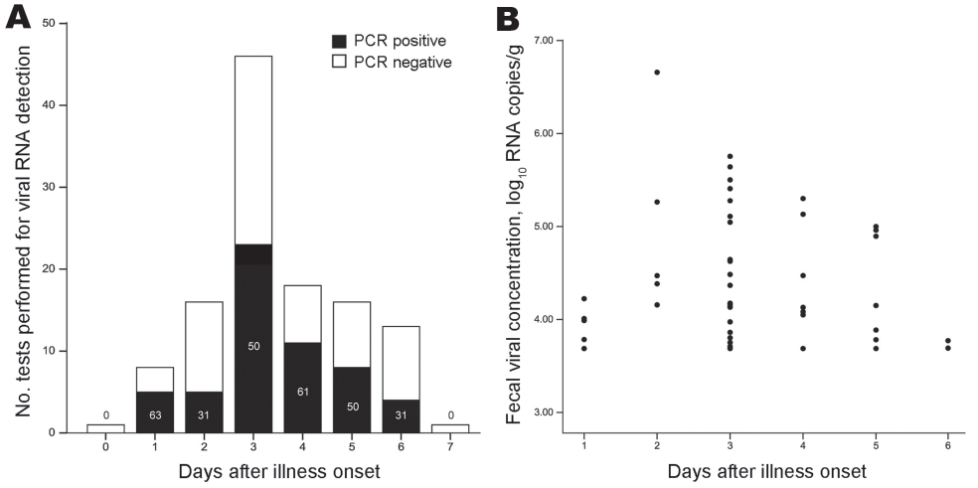
Fecal seasonal influenza A viral RNA detection rate and its concentration, by number of days after illness onset, Hong Kong, 2006–2009. A) Fecal viral RNA detection rate. Numbers in bars represent percentage of cases with positive viral RNA detection. B) Fecal viral RNA concentration. Three outliers were omitted from the figure for better illustration. Fecal viral RNA concentration was determined by using quantitative real-time reverse transcription PCR specific for the viral matrix gene and was expressed as log_10_ RNA copies/g of stool. The lower detection limit of the assay was 3.7 log_10_ RNA copies/g of stool.

A total of 119 hospitalized adults with seasonal influenza A infections were studied (Table 1). Their median age was 71 years (interquartile range [IQR] 57–79 years), and most (66%) had concurrent conditions; ≈5% were profoundly immunosuppressed. Vomiting and diarrhea were reported by 15 (13%) and 7 (6%) patients, respectively. Influenza A viral RNA was detected in 56 of 119 of stool samples, collected at a median interval of 3 days (IQR 3–5 days) from onset (detection rates by study year and virus subtype are shown in Table 2). Detection rate by day from onset ranged from 31% to 63% and showed a trend to decrease toward the end of the week (Figure 1, panel A). Overall, the mean ± SD fecal viral RNA concentration was 4.4 ± 0.8 log_10_ copies/g of feces and the median (IQR) was 4.2 (3.8–5.0) log_10_ copies/g of feces; concentrations tended to decrease with longer time elapsed from onset (Figure 1, panel B).

**Table 1:** Comparisons of baseline clinical and laboratory variables between influenza patients with positive and negative fecal viral RNA detection test results, Hong Kong, 2006–2009*

Patient characteristics	Fecal viral RNA–positive, n = 56	Fecal viral RNA–negative, n = 63	p value
Mean age, y (SD)	65.3 (18.6)	69.9 (13.4)	0.12
Age group, y, no. (%)			0.35†
18–49	7 (13)	6 (10)	
50–65	18 (32)	14 (22)	
>65	31 (55)	43 (68)	
Female sex, no. (%)	33 (59)	30 (48)	0.27
Interval from illness onset to sample collection <5 d, no. (%)	52 (93)	53 (84)	0.14
Concurrent condition, no. (%)			
Any	36 (64)	43 (68)	0.65
Major‡	31 (55)	37 (59)	0.72
Virus isolation, nasopharyngeal aspirates	50 (89)	49 (77)	0.06
Signs and symptoms when care was sought, no. (%)			
Fever	48 (86)	53 (84)	1.00
Cough and sputum	39 (74)	45 (76)	0.83
Sore throat	15 (28)	18 (31)	0.84
Rhinorrhea	21 (40)	21 (36)	0.70
Shortness of breath	18 (34)	27 (46)	0.25
Vomiting or diarrhea	10 (18)	9 (14)	0.63
Vomiting	7 (13)	8 (13)	1.00
Diarrhea	5 (9)	2 (3)	0.25
Laboratory parameters when care was sought			
Total leukocyte count, × 10^9^ cells/L, median (IQR)	7.3 (5.9–8.9)	7.9 (6.1–10.3)	0.18
Neutrophil count, × 10^9^ cells/L, median (IQR)	5.1 (4.2–7.0)	6.0 (4.1–7.7)	0.22
Lymphocyte count, × 10^9^ cells/L, median (IQR)	0.8 (0.6–1.1)	0.9 (0.6–1.2)	0.20
Lymphocyte count <1.0 × 10^9^ cells/L, no. (%)	37 (66)	29 (46)	0.03
Monocyte count, × 10^9^ cells/L, median (IQR)	0.7 (0.5–0.9)	0.7 (0.5–0.9)	0.51
Platelet count, × 10^9^ cells/L, median (IQR)	180 (142–228)	196 (156–248)	0.20
Alanine aminotransferase level, IU/L, median (IQR)	19 (13–32)	22 (15–36)	0.54
Antiviral treatment, no. (%)			
Oseltamivir§	35 (63)	42 (67)	0.70
Zanamivir	6 (11)	5 (8)	0.75
Specimen collected after starting antiviral drugs, no. (%)	39 (70)	42 (67)	0.84
Complication, no. (%)			
Any	39 (70)	44 (70)	0.98
Cardiorespiratory¶	29 (52)	38 (60)	0.36
Clinical outcome, no. (%)			
ICU admission	2 (4)	0 (0)	0.22
Death	0	0	NA
Median duration of hospitalization, d (IQR)	6 (4–12)	5 (4–13)	0.50

*Univariate comparisons of categorical and continuous variables were performed by using Fisher exact test and Mann-Whitney U test or Student *t* test, whenever appropriate. IQR, interquartile range; ICU, intensive care unit; NA, not applicable. †Fisher 2×3 exact test. ‡Defined as congestive heart failure; cerebrovascular, neoplastic, chronic liver, and renal diseases; diabetes; ischemic heart disease; or use of immunosuppressant drugs (*8*). Approximately 5% of patients were profoundly immunocompromised. §Oseltamivir (standard oral regimen, 75 mg 2×/d for 5 d). Amantadine was coadministered to 7 patients during the 2008–09 influenza season. ¶Defined as pneumonia, acute bronchitis, acute exacerbation of chronic respiratory conditions (e.g., chronic obstructive pulmonary disease, asthma); acute coronary syndrome, decompensated heart failure, arrhythmia, or acute cerebrovascular events (*8*).

**Table 2:** Fecal detection of seasonal influenza A viral RNA in stool samples, by year of study and virus subtype, Hong Kong, 2006–2009*

Year of study	No. fecal viral RNA–positive/ no. tested (%)	Fecal viral RNA–positive by virus subtype, no. (%)			Fecal viral RNA concentration by virus subtype, log_10_ RNA copies/g stool, median (IQR)	
		H1	H3		H1	H3
2006	11/20 (55)	7/7 (100)	2/4 (50)		5.0 (4.6–6.0)	NA
2007	19/35 (54)	0/0	19/31 (61)		NA	4.2 (4.0–5.1)
2008	11/26 (42)	1/5 (20)	10/21 (48)		NA	3.9 (3.7–4.4)
2009	15/38 (39)	5/10 (50)	10/27 (37)		3.9 (3.8–4.1)	3.9 (3.7–4.7)
All	56/119 (47)	13/22 (59)	41/83 (49)		4.6 (3.9–5.2)	4.2 (3.8–5.0)

*Virus subtyping results were unavailable for 9 cases in 2006, 4 cases in 2007, and 1 case in 2009. IQR, interquartile range; NA, not applicable.

In most (77%) viral RNA–positive samples, further H1- or H3-specific PCRs identified 7 cases as H1 and 36 cases as H3; unsuccessful amplification was associated with lower viral (matrix gene) concentrations (median [IQR] 3.9 [3.8–4.1] vs. 4.4 [3.8–5.1] log_10_ copies/g of stool; p = 0.04). No discrepancy was found between these and the subtyping results of the virus isolates from NPAs. Fecal viral RNA detection rate and concentrations were similar between H1 and H3 subtypes (Table 2).

Thirty-eight stool samples from 1 seasonal peak were subjected to virus isolation and viral RNA detection. In 10 cases, cytotoxicity occurred (procedure discontinued); in the remaining 28 cases, 12 were viral RNA positive; only 1 showed virus growth. This sample was from an 82-year-old man with dilated cardiomyopathy hospitalized for seasonal influenza A (H1N1) pneumonia and heart failure; diarrhea was absent.

Among 25 confirmed RSV or PIV infections (median [IQR] age 71 [55–79] years), viral RNA was detected in 5 fecal samples (collected at median [IQR] 4 [3–6] days after onset); none was culture positive. Fecal viral RNA positivity was lower compared with that of seasonal influenza viruses (p = 0.01).

Patients with positive and negative fecal viral RNA detection results were compared (Table 1). Positive fecal viral RNA detection was associated with younger age, shorter interval from illness onset to sample collection, lymphopenia, and positive virus isolation. Multivariate logistic regression showed that lymphopenia (adjusted odds ratio 2.36, 95% confidence interval 1.02–5.47; p = 0.045) and positive virus isolation in NPAs (adjusted odds ratio 3.76, 95% confidence interval 1.07–13.20; p = 0.039) were significant explanatory variables. No significant association was found between fecal viral RNA detection and clinical outcomes. Fecal viral RNA concentrations were also analyzed, and no association with clinical outcomes was found (data not shown), except for a negative correlation with lymphocyte count (Spearman ρ −0.37, p = 0.047).

**Figure 2:**
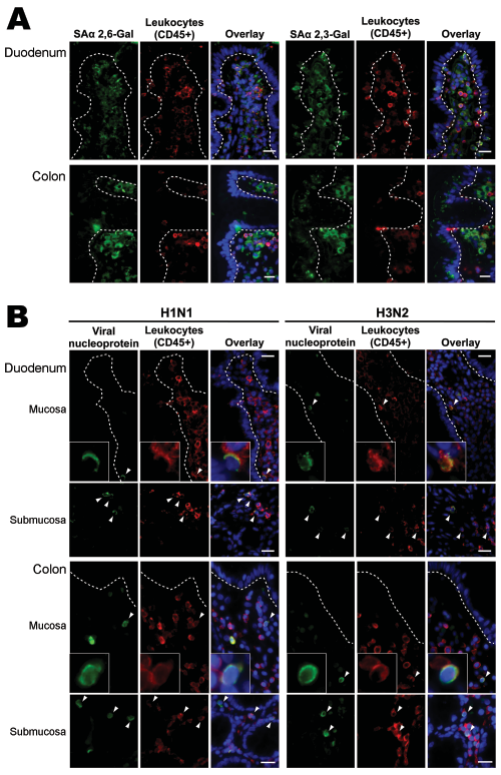
Intestinal distribution of influenza virus receptors and in vitro binding of inactivated seasonal influenza A (H1N1) and A (H3N2) viruses to human duodenal and colonic tissues. Images in the panels labeled Overlay show the green, red, and blue (nuclei counterstain) color channels in the same view. Dotted lines outline basal lining of intestinal epithelium. Arrowheads denote virus-bound cells. Scale bars = 20 µm. A) Double immunofluorescence staining showing that human-like influenza virus receptor sialic acid (SA) α 2,6-Gal; green) was not found on epithelial surface of small and large intestines but in lamina propria cells. Avian-like influenza virus receptor (SAα 2,3-Gal; green) was found on colonic epithelial surface and in lamina propria cells. Part of receptor-positive cells coexpressed CD45 (leukocyte common antigen; red), representing leukocytes. B) In vitro virus binding showing that neither seasonal influenza A (H1N1) nor A (H3N2) viruses bind to epithelial surface of small and large intestines but only to a subset of intestinal CD45+ leukocytes interspersed in the lamina propria and submucosa.

Lectin histochemical analysis showed that sialic acid α 2,6-Gal (human-like influenza virus receptor) was absent from epithelial surface of small and large intestines and was found only in lamina propria cells. In contrast, sialic acid α 2,3-Gal (avian-like influenza virus receptor) was found on large intestinal epithelial cells and in lamina propria cells. Virus-binding study showed that neither seasonal influenza A (H1N1) nor A (H3N2) virus bind to small and large intestinal epithelial surface, but they bind to a subset of CD45+ leukocytes (Figure 2).

## Conclusions

Direct intestinal infection by seasonal influenza viruses seems an unlikely explanation for the frequent fecal detection of viral RNA in the patients reported here. No clinical correlation was shown for RNA positivity (but was shown with lymphopenia and positive virus isolation in NPA, indicating higher virus load), and culture positivity is rare (*4**,**5**,**10**,**11*). Human-like influenza virus receptor is not found to express on normal intestinal epithelial cells (*12*). These findings agree with reports which showed that intestinal cells and tissues do not support efficient replication of seasonal viruses (*12**,**13*), thus their low potential to cause direct intestinal infection. Alternatively, swallowing of virus-containing nasopharyngeal secretions (although it seems inadequate to explain the higher rate of detecting fecal viral RNA than RSV or PIV) and hematogenous dissemination to organs through infected lymphocytes or macrophages in severe influenza cases with high virus load (spillover) are possible explanations for fecal viral RNA detection (*2**,**14*). Our findings on virus receptor distribution and in vitro virus binding to intestinal lamina propria leukocytes lends support to the latter hypothesis. Notably, viral RNA positivity in nonpulmonary tissues infiltrating mononuclear cells without detectable viral particles or antigens or tissue damage has been reported (*15*). Our study does not reject the possibility of seasonal influenza viruses causing occasional, disseminated infection in profoundly immunosuppressed persons because receptor affinity is not absolute (*2*). Conversely, highly pathogenic avian influenza (H5N1) and pandemic (H1N1) 2009 viruses have the ability to bind to avian-like influenza virus receptors on colonic epithelium and to replicate efficiently in intestinal cells and tissues (*12*). Their enhanced potential to cause direct intestinal infections and fecal–oral transmission deserve further investigation.

## Supplementary Material

Technical Appendix Technical details used in the fecal detection and isolation of viruses.
