# Supplementary material for: Seasonal Influenza A Virus in Feces of Hospitalized Adults
Source: Emerg Infect Dis. 2011 Nov;17(11):2038–42. doi: 10.3201/eid1711.110205 (PMC3310558; doi:10.3201/eid1711.110205)
Supplement: Technical Appendix — Technical details used in the fecal detection and isolation of viruses. [file 11-0205-Techapp_4p.pdf]

Article DOI: http://dx.doi.org/10.3201/eid1711.110205

Seasonal Influenza A Virus in Feces of Hospitalized Adults

Technical Appendix

Fecal Detection and Isolation of Influenza A Virus

Detection and quantification of fecal influenza A viral RNA were performed by quantitative real-time reverse transcription PCR (RT-PCR) specific for the viral matrix-gene as described (*1–3*). Briefly, viral RNA was extracted (in batches of 5–20 frozen [−80°C] specimens) from a 10% stool suspension in phosphate-buffered saline (100 mg of stool plus 900 µL of saline) by using the QIAamp Viral RNA Mini Kit (QIAGEN, Valencia, CA, USA). Synthesis of complementary DNA from purified RNA was performed by using M-MLV reverse transcriptase (Promega, Madison, WI, USA) and random hexamers. Fecal viral RNA concentration was determined against 10-fold serially diluted plasmid standards prepared from cloning the amplicon into pCR 2.1-TOPO vector (Invitrogen, Carlsbad, CA, USA). The lower limit of detection of the assay is 3.7 log_{10} RNA copies per gram of stool (log_{10} copies/g stool). In addition, virus subtyping was performed on the viral RNA–positive specimens by using H1- and H3-specific conventional PCRs as described (*4*).

To minimize specimen contamination, aerosol-resistant tips were used in all liquid pipetting throughout the experiments; negative controls without stool in viral RNA extraction and negative controls without template in RT-PCR steps were included in every run; and PCR master mixture preparation, template addition, and PCR were performed in 3 separate, designated areas. During PCR setup, reaction tubes containing specimen materials were capped before adding plasmid standards to control tubes. No false-positive results were observed in any negative controls.

Fresh stool specimens collected from influenza patients during 1 seasonal peak were simultaneously subjected to virus isolation. Briefly, a 10% stool suspension in viral transport medium was prepared immediately after specimen collection. After centrifugation, the suspension was sterilized through a 0.45-µm filter and adjusted to physiologic pH range. MDCK

cells were then inoculated with the stool filtrate at 37°C for 1 hour, rinsed, and incubated at 33°C on a roller drum for 10–14 days. Cells were monitored daily for evidence of cytotoxicity and cytopathic effect. Detection of viral antigen was performed by using an immunofluorescence assay.

Fecal Detection and Isolation of Respiratory Syncytial Virus and Parainfluenza Virus

An approach similar to that of seasonal influenza virus detection was used for fecal virus detection among respiratory syncytial virus (RSV)– or parainfluenza virus (PIV)–infected patients. Briefly, stool specimens were processed as described above. Detection of fecal viral RNA was performed by using TaqMan-based RT-PCRs specific for the nucleocapsid gene of RSV and hemagglutinin-neuraminidase genes of PIV (*5*). Virus isolation was performed by using HEp-2 cells for RSV and LLC cells for PIV. Viral antigen was detected by virus-specific immunofluorescence assay.

Double Immunofluoresence Staining for Influenza Virus Receptors and Surface Marker of Receptor-positive Cells in Human Small and Large Intestinal Tissues

Archived formalin-fixed, paraffin-embedded human adult normal duodenal and colonic biopsy tissues obtained from 6 persons (3 duodenal and 3 colonic, all without influenza infection) were used for virus receptors distribution study. Sections of 5-µm thickness were prepared from tissue blocks, deparaffinized in xylene, and rehydrated in sequential ethanol gradient (from 100% to 70%). Antigen retrieval was performed by boiling the sections in 10 mmol/L citrate buffer for 15 minutes. After cooling, sections were incubated with Image-iT FX signal enhancer (Invitrogen) for 30 minutes, followed by protein block (Dako, Roskilde, Denmark) for 30 minutes and avidin/biotin blocking kit reagents (Vector Laboratories, Burlingame, CA, USA) as per manufacturer's instruction.

Sections were double-labeled by incubating with 10 µg/mL of biotinylated *Sambucus nigra* lectin (against human influenza virus receptor sialic acid α-2,6 galactose) or *Maackia amurensis* lectin I (against avian influenza virus receptor sialic acid α-2,3 galactose; Vector Laboratories), and mouse anti-human monoclonal antibody against CD45 (leukocyte common antigen) (Dako) at 4°C overnight (14–18 hours). Sections were then incubated with 2 µg/mL of Alexa Fluor 488–conjugated streptavidin (Invitrogen) and Alexa Fluor 594–conjugated goat anti-mouse immunoglobulin (Invitrogen) for 60 minutes, and mounted with ProLong Gold antifade

reagent containing 4′,6-diamidino-2-phenylindole (Invitrogen). Images of each color channel were captured by using epifluorescence microscope. To test the specificity of the staining, negative controls using protein block reagent in replacement of the lectin, and isotype-matched control in replacement of the primary antibody were included. Neighboring sections were used for the controls. Sections were washed with phosphate-buffered saline with or without Tween20 between steps, where appropriate. All steps were performed at room temperature unless otherwise specified.

In Vitro Binding of Influenza A Viruses to Human Small and Large Intestinal Tissues

Human isolates of seasonal influenza A H1N1(A/HongKong/CUHK-13003/2002) and H3N2(A/HongKong/CUHK-22910/2004) viruses were propagated in MDCK cells, and virus-containing supernatants were inactivated in 0.2% formalin for at least 3 days. Inactivated viruses were concentrated by using Amicon Ultra-4 (100 kDa) centrifugal filter unit (Millipore) and then dialyzed against phosphate-buffered saline. In vitro virus binding was performed on formalin-fixed, paraffin-embedded tissues. Tissue sections were processed as described above and then incubated with the viruses (3×10^4 RNA copies) at 4°C overnight. Monoclonal antibodies against viral nucleoprotein (clone A1 for subtype H1N1 and clone A3 for subtype H3N2, 10 µg/mL; Millipore, Billerica, MA, USA) were used to detect the viruses. Double immunofluorescence staining for the viruses and CD45 was performed by using the Vector M.O.M. system as per manufacturer's instructions (Vector Laboratories). Signals were visualized by using Alexa Fluor 488 for viral nucleoprotein and Alexa Fluor 594 for CD45.

References

1. Chan MC, Lee N, Chan PK, Leung TF, Sung JJ. Fecal detection of influenza A virus in patients with concurrent respiratory and gastrointestinal symptoms. J Clin Virol. 2009;45:208–11. PubMed doi:10.1016/j.jcv.2009.06.011

2. Chan MC, Sung JJ, Lam RK, Chan PK, Lai RW, Leung WK. Sapovirus detection by quantitative real-time RT-PCR in clinical stool specimens. J Virol Methods. 2006;134:146–53. PubMed doi:10.1016/j.jviromet.2005.12.013

3. Lee N, Wong CK, Chan PK, Lun SW, Lui G, Wong B, et al. Hypercytokinemia and hyperactivation of phospho-p38 mitogen-activated protein kinase in severe human influenza A virus infection. Clin Infect Dis. 2007;45:723–31. PubMed doi:10.1086/520981

4. Chi XS, Li F, Tam JS, Rappaport R, Cheng SM. Semiquantitative one-step RT-PCR for simultaneous identification of human influenza and respiratory syncytial viruses. J Virol Methods. 2007;139:90–2. PubMed doi:10.1016/j.jviromet.2006.08.015

5. van de Pol AC, van Loon AM, Wolfs TF, Jansen NJ, Nijhuis M, Breteler EK, et al. Increased detection of respiratory syncytial virus, influenza viruses, parainfluenza viruses, and adenoviruses with real-time PCR in samples from patients with respiratory symptoms. J Clin Microbiol. 2007;45:2260–2. PubMed doi:10.1128/JCM.00848-07
